# Characterization of 10-Hydroxygeraniol Dehydrogenase from *Catharanthus roseus* Reveals Cascaded Enzymatic Activity in Iridoid Biosynthesis

**DOI:** 10.1038/srep08258

**Published:** 2015-02-05

**Authors:** Ramakrishnan Krithika, Prabhakar Lal Srivastava, Bajaj Rani, Swati P. Kolet, Manojkumar Chopade, Mantri Soniya, Hirekodathakallu V. Thulasiram

**Affiliations:** 1 Chemical Biology Unit, Division of Organic Chemistry, CSIR- National Chemical Laboratory, Dr. Homi Bhabha Road, Pune 411008; 2 CSIR-Institute of Genomics and Integrative Biology, Mall Road, New Delhi 110007

## Abstract

*Catharanthus roseus* [L.] is a major source of the monoterpene indole alkaloids (MIAs), which are of significant interest due to their therapeutic value. These molecules are formed through an intermediate, *cis*-*trans*-nepetalactol, a cyclized product of 10-oxogeranial. One of the key enzymes involved in the biosynthesis of MIAs is an NAD(P)^+^ dependent oxidoreductase system, 10-hydroxygeraniol dehydrogenase (Cr10HGO), which catalyses the formation of 10-oxogeranial from 10-hydroxygeraniol via 10-oxogeraniol or 10-hydroxygeranial. This work describes the cloning and functional characterization of Cr10HGO from *C. roseus* and its role in the iridoid biosynthesis. Substrate specificity studies indicated that, Cr10HGO has good activity on substrates such as 10-hydroxygeraniol, 10-oxogeraniol or 10-hydroxygeranial over monohydroxy linear terpene derivatives. Further it was observed that incubation of 10-hydroxygeraniol with Cr10HGO and iridoid synthase (CrIDS) in the presence of NADP^+^ yielded a major metabolite, which was characterized as (1*R*, 4a*S*, 7*S*, 7a*R*)-nepetalactol by comparing its retention time, mass fragmentation pattern, and co-injection studies with that of the synthesized compound. These results indicate that there is concerted activity of Cr10HGO with iridoid synthase in the formation of (1*R*, 4a*S*, 7*S*, 7a*R*)-nepetalactol, an important intermediate in iridoid biosynthesis.

Monoterpene indole alkaloids (MIAs) are a multifarious class of natural products with distinct chemical and biological properties^1^,^2^,^3^. To date, over 3000 MIAs are known with diverse structures and biological activities. The Apocynaceae family plant, *C. roseus* is a rich source of the iridoid-derived MIAs and is known to contain over 200 alkaloids in various tissues. Two MIAs from this plant, vincristine and vinblastine, are widely prescribed as potent anti-cancer agents^4^,^5^. These MIAs were synthesized from the condensation of tryptamine and the iridoid monoterpene, secologanin. The MIAs' biosynthesis diverges from the isoprenoid biosynthetic pathway at the 1′-4 chain elongation intermediate geranyl diphosphate (GPP) formed through head-to-tail condensation of isopentenyl diphosphate (IPP) with dimethylallyl diphsophate (DMAPP) catalyzed by Geranyl diphosphate synthase (CrGDS)^6^. Geraniol synthase (CrGS)^7^ hydrolyses GPP into geraniol, which undergoes hydroxylation at C10 to form 10-hydroxygeraniol by the cytochrome P450 system, Geraniol 10-hydroxylase (CrG10H)^8^ (Fig. 1). Feeding experiments with labelled 10-hydroxygeraniol, 10-hydroxynerol and iridodiol in *C. roseus* and *Lonicera morrowii* suspension cultures clearly indicated that 10-hydroxygeraniol is oxidized to 10-oxogeranial by the oxidoreductase system, 10-hydroxygeraniol dehydrogenase (10HGO)^9^,^10^,^11^,^12^ (Fig. 1). Recently, a short chain reductive cyclase, iridoid synthase (CrIDS)^13^, which cyclises 10-oxogeranial into an equilibrium mixture of *cis*-*trans*-nepetalactol and iridodials, has been characterized. The bicyclic compound, *cis*-*trans*-nepetalactol, is the key intermediate involved in the biosynthesis of a structurally diverse array of MIAs. Experiments using labelled intermediates indicated that one of the committed steps during the biosynthesis of iridoids is the oxidation of 10-hydroxygeraniol to its dialdehyde cognate, 10-oxogeranial^11^. Ikeda *et al*.^14^ had purified the NADP^+^ dependent oxidoreductase protein from *Rauwolfia serpentina* cells which could convert 10-hydroxygeraniol into 10-oxogeraniol, 10-hydroxygeranial and 10-oxogeranial. However, it was found to have better activity on nerol and geraniol. The present work describes the cloning and functional characterization of Cr10HGO and the study on the orchestration of enzyme activity of Cr10HGO with CrIDS in the biosynthesis of desired (1*R*, 4a*S*, 7*S*, 7a*R*)*-*nepetalactol (Fig. 1). Also, substrate specificity studies of Cr10HGO indicated that, it has broad substrate specificity with 10-hydroxygeraniol, 10-oxogeraniol or 10-hydroxygeranial over monohydroxy linear terpene derivatives.

## Results and Discussion

### Transcriptome Analysis

RNA sequencing is a powerful technique for profiling transcriptome because of its high-throughput accuracy and reproducibility. In plants, high-throughput RNA sequencing has accelerated the discovery of novel genes, transcription pattern, and functional analysis. In the present work, we have performed RNA sequencing on Illumina GAII Analyzer, and have screened for unigenes involved in the biosynthesis of secologanin in *Catharanthus roseus*. The raw RNA-seq paired reads from stem, root and leaf RNA were deposited with NCBI (Accession ID: SRR1693842) and have been assembled using the Velvet_1.1.05 (Oases).

Various approaches for functional annotation of the assembled transcripts have been used to identify the genes, which are involved in MIA biosynthesis in *C. roseus*. All the 62,352 putative unigenes obtained were compared with manually curated KEGG (Kyoto Encyclopedia of Genes and Genomes) database of *Arabidopsis thaliana* (Thale cress) and *Oryza sativa japonica* (Japanese rice) for functional annotation of genes by bidirectional BLAST. 4335 unigenes were assigned with KEGG Orthology (KO) number representing 327 KEGG pathways involved in majority of plant biochemical pathways including metabolism, cellular processes and genetic information processing. All the unique transcripts (62,352) were submitted to Virtual Ribosome-V1.1 to predict ORF of maximum length for each unigene in all six reading frames. A total of 62,290 unigenes (99.9%) were identified as having an ORF starting at the ATG codon, from which 22,224 unigenes (35.64%) contained the ORF of ≥70 amino acids length. To identify protein domain architecture, these 22,224 unigenes were submitted for Pfam analysis against the PfamA database. The transcripts for various Pfam domain matches were identified and run individually on BLAST program to identify various ORFs related to the mevalonate (MVA) pathway, methylerythritol phosphate (MEP) pathway and Secologanin biosynthetic pathway. A summary of the ORFs identified is shown in Supplementary Table S1.

### Cloning and functional characterization of Cr10HGO

An Open reading frame (ORF) of 1083 bp (Supplementary Fig. S1), encoding a polypeptide of 360 amino acids (Supplementary Fig. S2), displaying 78% sequence identity with both cinnamyl alcohol dehydrogenase from *Populus trichocarpa* (Genbank ID: ACC63874^15^) and geraniol dehydrogenase from *Ocimum basilicum* (GenBank ID: Q2KNL6^16^), was identified as Cr10HGO (KF561458). Deduced amino acid sequence of Cr10HGO was found to have a calculated molecular weight of 38.9 kDa, comprising zinc binding alcohol dehydrogenase signature sequence (positions 71–85) with the PROSITE ID: ADH_ZINC (PS00059), and the consensus sequence G-H-E-x-EL-G-7-x(4)-[GA]-x(2)-[IVSAC]. Although the sequence of this gene was shown to be similar to the gene AY352047^17^ found in the data bank, its functional characterization has not been published. This unigene was cloned in pRSET B expression vector with N-terminal His_6_-tag under the control of T7-RNA polymerase promoter for expression of soluble active proteins in *E. coli* BL21 (DE3) cells. Recombinant His_6_-tagged proteins were purified to homogeneity by Ni^2+^-affinity chromatography with the yield of 12 mg/L (Supplementary Fig. S2).

**Figure 2:**
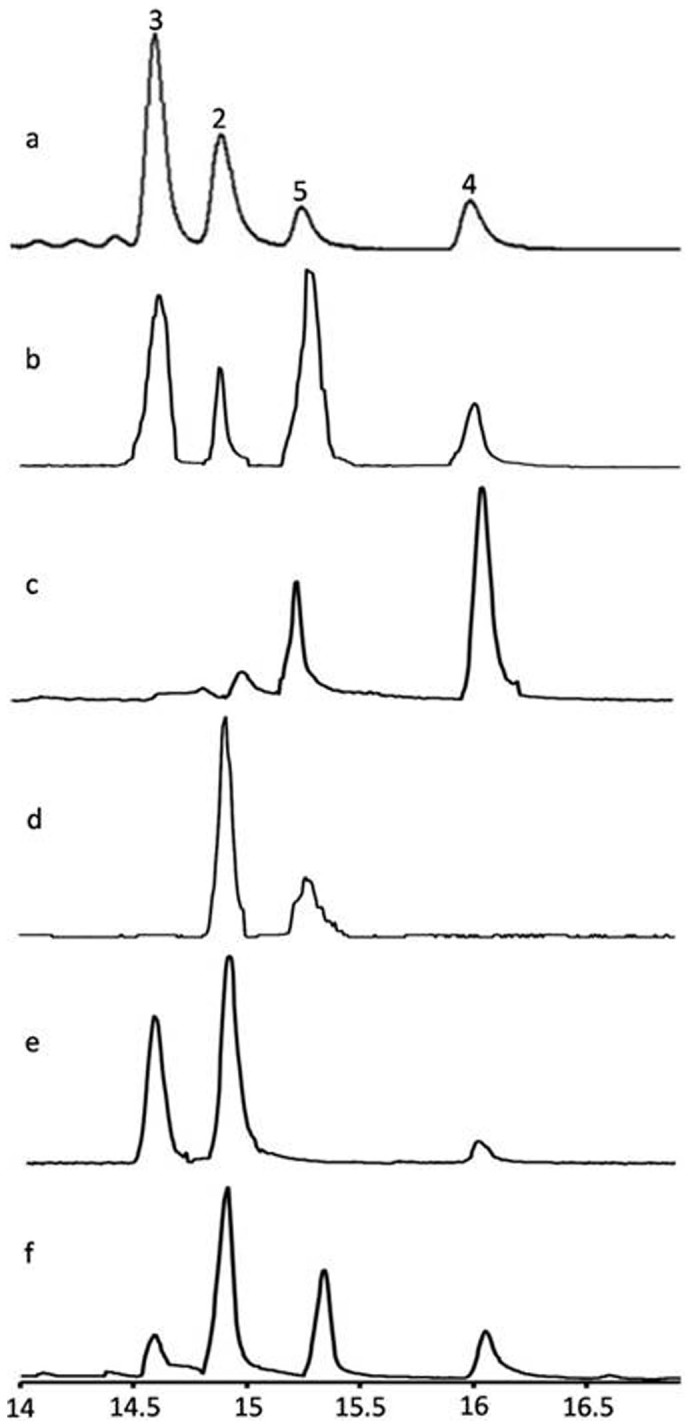
Total ion chromatograms (TICs) for 10-hydroxygeraniol dehydrogenase (Cr10HGO) catalyzed reactions with (a) 10-hydroxygeraniol and NADP^+^, (b) 10-oxogeraniol and NADP^+^, (c) 10-oxogeraniol and NADPH, (d)10-oxogeranial and NADPH, (e) 10-hydroxygeranial and NADP^+^, (f) 10-hydroxygeranial and NADPH as substrates. The peaks represent (**2**) 10-hydroxygeraniol, (**3**) 10-oxogeraniol, (**4**) 10-hydroxygeranial, and (**5**) 10-oxogeranial.

**Figure 3:**
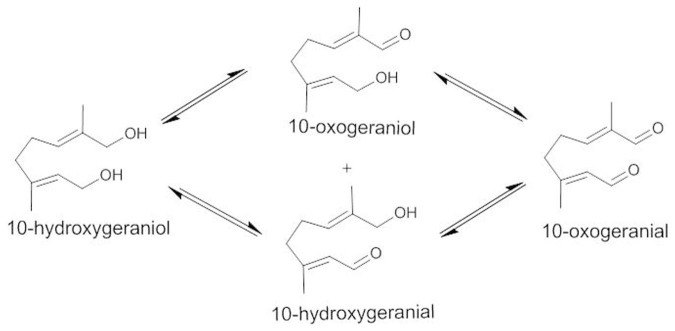
10-hydroxygeraniol dehydrogenase (Cr10HGO) catalyzed reaction. NADP^+^ is utilized in the forward reaction, whereas NADPH is utilized in the reverse reactions, generating vice-versa in the process.

Gas chromatography and mass spectrometric analyses of the reaction products after incubation of purified Cr10HGO protein with 10-hydroxygeraniol in the presence of NADP^+^ resulted in the formation of 10-oxogeranial along with 10-oxogeraniol and 10-hydroxygeranial as minor products (Fig. 2a and Supplementary Fig. S3). The formation of these products was further confirmed by comparing the fragmentation pattern as well as co-injection studies using corresponding synthesized compounds, 10-oxogeranial, 10-oxogeraniol and 10-hydroxygeranial. Further, Cr10HGO efficiently converted 10-oxogeraniol and 10-hydroxygeranial into 10-oxogeranial in the presence of NADP^+^. However, when NADPH was used as cofactor, 10-hydroxygeraniol was found to be the major enzymatic product with substrates, 10-oxogeraniol, 10-hydroxygeranial and 10-oxogeranial (Fig. 2) indicating that the Cr10HGO mediated reaction (Fig. 3) is reversible. The NADP^+^ dependent oxidoreductase protein purified from *R. serpentina* catalyzes dehydrogenation of nerol and geraniol in an efficient manner compared to10-hydroxygeraniol^14^. Similarly, the oxidoreductase purified from *Nepeta racemosa*^18^ also showed better activity towards geraniol, nerol and 10-hydroxynerol than towards 10-hydroxygeraniol. The recently reported 8-HGO, which encodes the NADP^+^ dependent oxidoreductase from *C. roseus* carries out the dehydrogenation of 10-hydroxygeraniol and also other acyclic monoterpenes^19^, but does not possess much sequence similarity with Cr10HGO. In contrast to these observations, monohydroxy terpene derivatives such as geraniol, nerol, and farnesol were found to be poor substrates for Cr10HGO as compared to the reported 8HGO^19^ (Supplementary Table S2 and Supplementary Fig. S4). Studies on the effects of temperature on Cr10HGO mediated reaction revealed that the Cr10HGO activity was found to be optimum at 30°C. The apparent K_m_ values were found to be 1.50 μM for 10-hydroxygeraniol, 1.0 μM for 10-oxogeraniol and 10-hydroxygeranial at saturated concentrations of NADP^+^ (Supplementary Figures S5–S11, Supplementary Table S3). The kinetic studies also indicated that among NAD^+^/(H) and NADP^+^/(H), the latter was found to be the preferred coenzyme for Cr10HGO (Table 1).

### Cloning and functional characterization of CrIDS

**Figure 4:**
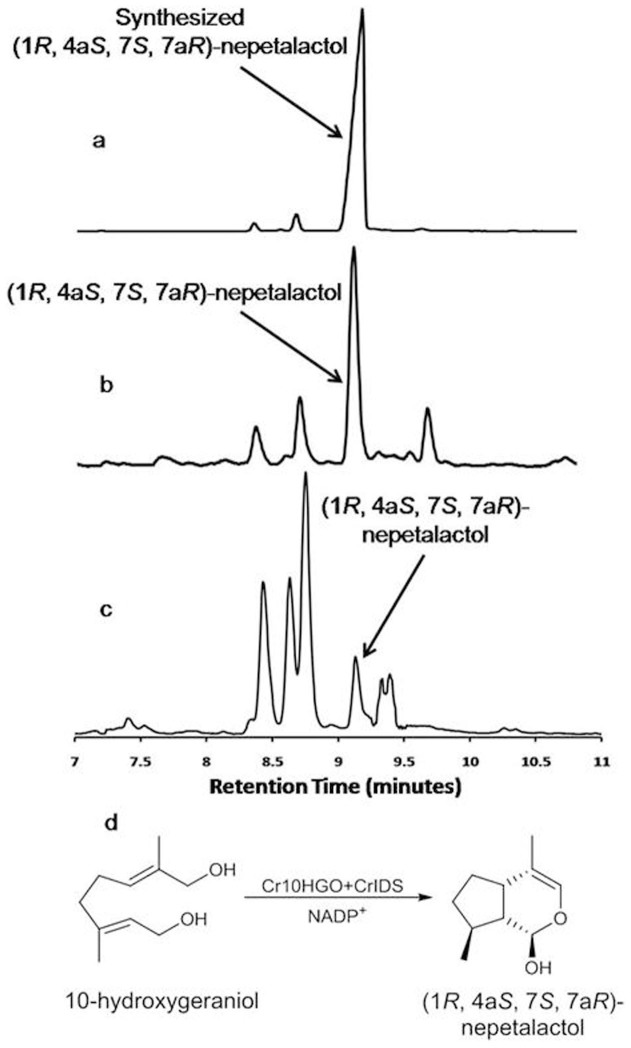
Comparison of TICs of (a) Synthesized (1*R*, 4a*S*, 7*S*, 7a*R*)-nepetalactol, (b) Extract of combined assay of Cr10HGO and CrIDS with 10-hydroxygeraniol and NADP^+^ as substrates, and (c) Extract of CrIDS assay with 10-oxogeranial and NADPH as substrates. (d) Concerted reaction of Cr10HGO and CrIDS on 10-hydroxygeraniol to form (1*R*, 4a*S*, 7*S*, 7a*R*)-nepetalactol.

Full length cDNA sequence of CrIDS unigene was obtained by using the primers designed from the transcript number 2280. The ORF of CrIDS (KF56146) composed of 1161 bp (Supplementary Fig. S12) encoding a polypeptide of 386 amino acids (Supplementary Fig. S13), with a calculated molecular weight of 43.69 KDa. This polypeptide sequence showed high similarity with the reported IDS sequence but was shorter by six nucleotides (nucleotides from 47–52 missing), thereby causing a deletion of two amino acids (Pro and Asn). Nevertheless, this shorter CrIDS efficiently carried out the reductive cyclization of 10-oxogeranial into four compounds (Supplementary Fig. S14). The GC and GC-MS analyses of the assay mixture extracts of 10-oxogeranial with CrIDS, in the presence of NADPH, indicated the presence of an equilibrium mixture of *cis*-*trans*-nepetalactol and iridodials (Rt: 8.5, 8.7, 8.8 and 9.2 min) (Fig. 4c), in line with the other study^13^.

### Cascaded enzyme activity of Cr10HGO with CrIDS

Surprisingly, when 10-hydroxygeraniol was incubated with Cr10HGO and CrIDS for 30 min, in the presence of NADP^+^, it yielded a major metabolite (Rt: 9.2 min) (Fig. 4b). The major metabolite formed was identified as *cis-trans*-nepetalactol with stereochemistry 4a*S*, 7*S*, 7a*R* by comparing the retention time and co-injection studies in GC and GC-MS analyses with the synthesized diastereomeric mixture of *cis-trans*-nepatalactols [containing (1*R*, 4a*S*, 7*S*, 7a*R*)*-*nepetalactol as a major diastereomer]^20^,^21^,^22^ arising due to the asymmetry at carbon 1. These two diastereomers were not resolved under the GC conditions with chiral capillary column. However, the acetylated diastereomers were separated under similar GC conditions (Supplementary Fig. S15). The stereochemistry of the major enzymatic product formed was determined as (1*R*, 4a*S*, 7*S*, 7a*R*)*-*nepetalactol acetylation of assay mixture, followed by GC and GC-MS analyses and comparing the retention time and co-injection studies with that of acetylated derivatives of synthesized nepetalactols' mixture containing (1*R*, 4a*S*, 7*S*, 7a*R*)*-*nepetalactol as a major diastereomer^21^ (Supplementary Figures S15, S16 and S17).

Further, when 10-hydroxygeraniol was incubated with Cr10HGO and CrIDS in the presence of NADP^+^ for prolonged incubation beyond 30 min led to the formation of open structures of iridodials in equilibrium with *cis*-*trans*-nepetalactol (Supplementary Fig. S18). The formation of a major metabolite by the combined action of two enzymes clearly indicates the concerted enzymatic action of Cr10HGO and CrIDS in the formation of desired (1*R*, 4a*S*, 7*S*, 7a*R*)-nepetalactol, an important intermediate in iridoids and MIAs biosynthesis. As both Cr10HGO and CrIDS are cytoplasmic enzymes, presumably, the products of Cr10HGO [10-oxogeranial and NAD(P)H] will be used by CrIDS to synthesize (1*R*, 4a*S*, 7*S*, 7a*R*)*-*nepetalactol, indicating a physiological enzyme cascade.

### Cloning and Functional Characterization of CrGDS, CrGS and CrG10H

As a part of ongoing efforts to elucidate the MIAs biosynthetic pathway in *C. roseus* and also to explore their production in heterologous systems, the full length unigenes, which showed high ranking with known GDS and GS from various sources, were used for the cloning and functional characterization of CrGDS and CrGS from *C. roseus*. CrG10H was cloned using the primers designed from the reported gene, which encodes geraniol hydroxylase in *C. roseus*^23^ as the unigenes 742 had similar sequence to that of the reported one (Supplementary Table S4). These genes were cloned in various expression vectors compatible with *E. coli* or yeast systems and the expressed proteins were purified by Ni-NTA chromatography, except CrG10H, which remained as microsomal pellet when expressed in yeast system (Supplementary Figures S19–S27). While we were characterizing CrGDS and CrGS from *C. roseus*, similar studies were reported elsewhere^6^,^7^.

To understand the combinatorial action of these enzymes on their cognate substrates, we incubated dimethylallyl diphosphate (DMAPP) and isopentenyl diphosphate (IPP) with purified CrGDS and CrGS proteins and yeast expressed microsomal pellet containing CrG10H, in the presence of coenzymes, which yielded 10-hydroxygeraniol as the enzymatic product (Supplementary Figures S28 and S29). Furthermore, incubation of geraniol in combination with CrG10H, Cr10HGO and CrIDS revealed the formation of (1*R*, 4a*S*, 7*S*, 7a*R*)*-*nepetalactol by GC and GC-MS analyses (Supplementary Figures S30 and S31).

These experiments suggest *in vivo* proximity and “cross talk” of the enzymes involved in the biosynthetic cascade leading to the formation of the desired product. As CrG10H is localized on endoplasmic reticulum, the product of this enzyme, 10-hydroxygeraniol might be sequestered out to the subsequent enzymes of MIAs pathway. These observations are further supported by the studies on cellular localization of MIAs biosynthetic pathway enzymes in a particular cell type in *C. roseus*^24^,^25^,^26^,^27^. It also appears that the formation of 10-hydroxygeraniol through CrG10H catalyzed reaction is the rate-limiting step for iridoid biosynthesis.

### Concluding Remarks

Cloning and functional characterization of 10-hydroxygeraniol dehydrogenase (Cr10HGO) system from *C. roseus* indicated that Cr10HGO showed broad substrate specificity for 10-hydroxygeraniol, 10-oxogeraniol or 10-hydroxygeranial over monohydroxy linear terpene derivatives. Concerted enzymatic function in the biosynthesis of *cis*-*trans*-nepetalactol has been demonstrated using 10-hydroxygeraniol and NADP^+^ with Cr10HGO and CrIDS combined assay system. The stereochemistry of the enzymatic product was determined and is (1*R*, 4a*S*, 7*S*, 7a*R*)-nepetalactol, which is a key intermediate in the biosynthesis of iridoids and MIAs. Further we have demonstrated the *in vitro* formation of (1*R*, 4a*S*, 7*S*, 7a*R*)-nepetalactol when geraniol was incubated with CrG10H, Cr10HGO and CrIDS.

## Methods

### Plant Sample Source and Strains used

The various tissues, leaves, stem and roots were collected from *C. roseus* plants of height 26 cm above the ground, grown in a greenhouse. The tissues were flash frozen in liquid nitrogen and stored in −80°C till used. A summary of the strains and plasmids used in the study are mentioned in Supplementary Table S4.

### Isolation of Total RNA and cDNA Synthesis

Total RNA was extracted from the leaf, stem and root tissues of *C. rosues* using Spectrum™ Plant Total RNA Isolation Kit from Sigma (Supplementary Fig. S32). These RNA samples were utilised for Transcriptome Sequencing (all combined in 1:1 ratios), and for construction of cDNA using SuperScript III RT Kit from Invitrogen.

### Transcriptome Sequencing and Assembly

Briefly, mRNA was purified from 1 μg of intact total RNA using oligodT beads (TruSeq RNA Sample Preparation Kit, Illumina). The purified mRNA was fragmented for 2 minutes at elevated temperature (94°C) in the presence of divalent cations and reverse transcribed with Superscript II Reverse transcriptase by priming with Random Hexamers. Second strand cDNA was synthesized in the presence of DNA polymerase I and RnaseH. The cDNA was cleaned up using Agencourt Ampure XP SPRI beads (Beckman Coulter). Illumina Adapters were ligated to the cDNA molecules after end repair and addition of a base. SPRI cleanup was performed after ligation. The library was amplified using 8 cycles of PCR for enrichment of adapter ligated fragments. The prepared library was quantified using Nanodrop and validated for quality by running an aliquot on High Sensitivity Bioanalyzer Chip (Agilent).

A total of 15.49 million raw reads were generated with a length of 70 bp. Adapter trimming and low quality trimming was performed throughout the sequence to get better quality reads. High quality reads (>20 phred score) were then used for *de novo* assembly with varying hash lengths. The 12,409,039 raw reads (80.11%) obtained were assembled into 53,544 contigs with optimized hash length of 49, having an average contig length of 1594.83 bp and N50 value of 2485. These contigs were submitted as inputs for Oasis_0.2.01 to generate 70,779 transcripts having N50 value of 2355 and an average transcript length of 1457.98 bp. These transcripts were further subjected to cluster and assembly analysis using CD-HIT to remove the redundancy, which resulted in a total of 62,352 unique transcripts with an average size of 1024 bp and N50 value of 2375.

### Sequence Analysis

Analysis of sequenced samples was carried out using NCBI's BLAST tool (http://blast.ncbi.nlm.nih.gov/Blast.cgi). Theoretical molecular weight was calculated using Protein Molecular Weight Calculator tool (http://www.bioinformatics.org/sms/prot_mw.html).

### Cloning and Expression of Cr10HGO, CrGDS, CrGS, CrG10H and CrIDS

The full length transcripts which showed high ranking with known GDS, GS, G10H, 10HGO and the reductive cyclization enzymes from various sources were used for the cloning and expressed in suitable heterologous systems (Supplementary Tables S5 and S6).

### Yeast Expression and Microsome Preparation (CrG10H)

Expression of active protein was carried out in INVSc1 yeast competent cells. Cells were grown overnight in synthetic complete medium without Uracil (SC-U), containing 2% glucose at 30°C, then transferred to induction medium (SC-U, containing 20% galactose) and further incubated at 30°C for 12 hours. The cells were centrifuged at 3000 × *g* for 10 minutes at 4°C. The cell pellet obtained was washed with TEK buffer (50 mM Tris-HCl, 1 mM EDTA, pH 7.4, 100 mM KCl) (1 mL/g of cell pellet × 3) and centrifuged. The cell pellet (1 g/5 mL) was re-suspended in 50 mM Tris-HCl buffer (containing 1 mM EDTA, 600 mM Sorbitol, 5 mM DTT, 0.25 mM PMSF and pH 7.4) and cells were lysed using a bead-beater (with acid washed glass beads, 425–600 μm) for 6 cycles (pulse on 30 sec, pulse off 30 sec, manual rocking for 3 × 30 sec). The lysed cells were centrifuged at 1000 × *g* for 5 min at 4°C to remove the glass beads. Further, the supernatant was subjected to centrifugation at 10,000 × *g* for 30 min at 4°C. The 10,000 × *g* supernatant was centrifuged at 1,00,000 × *g* for 1 hr 30 min at 4°C. The microsomal pellet, thus obtained, was suspended in TEG buffer (50 mM Tris-HCl, 1 mM EDTA, 30% glycerol, pH 7.5) and homogenized. The homogenized microsome fraction was aliquoted (0.2 mL), flash-frozen in liquid nitrogen and stored at −80°C.

### Chemical Synthesis

Geraniol acetate, Citral, 10-oxonerayl acetate and (*S*)-citronellol were purchased from Sigma-Aldrich. The compounds **2**, **3**, **4**, **5**, **12** and **16** (Supplementary Schemes S1 and S2) were synthesized according to the procedures described earlier^21^,^22^. Nepetalactol (**11**) was prepared according to the procedure described by Beckett *et. al*.^20^ **4** was found to contain a mixture of ~24% of **3**. All the spectral data recorded for these compounds were in accordance with those obtained in the literature (Supplementary Figures S35–S52).

### Product Ratio Studies of Cr10HGO

To 0.1 mg of protein in 0.5 mL of sodium bicarbonate buffer (20 mM sodium bicarbonate, 10% v/v glycerol, pH 10.0), containing NADP^+^, 0.2 mM of 10-hydroxygeraniol was added and the mixture incubated at 30°C for 30 minutes. The mixture was then extracted thrice with dichloromethane (CH_2_Cl_2_). The combined organic phase was dried over sodium sulphate, reduced to ~50 μL with a stream of dry nitrogen and subjected to GC and GC-MS analyses. Assays with various other substrates (Supplementary Table S2) were carried out with Cr10HGO under identical conditions.

### Determination of Kinetic Parameters

Steady-state kinetics was performed in 20 mM Sodium bi-carbonate, 10% v/v Glycerol, pH 10.0 at 30°C with varying substrate concentrations, ranging from 0.25 to 500.0 μM with saturation concentration of cofactor, NADP^+^ (500.0 μM) and vice versa. The reactions were followed by measuring changes in NADPH concentration at 340 nm. The kinetic data were fitted with the Graph Pad Prism software and the parameters calculated using Michaelis-Menten plots. Similarly, kinetic parameters for 10-hydroxygeraniol (with NAD^+^), 10-oxogeraniol (with NADP^+^ or NADPH), 10-oxogeranial (with NADPH), 10-hydroxygeranial (with NADP^+^ or NADPH) and 10-hydroxynerol (with NADP^+^) were determined.

### Combined Assays

The combined assay of CrGDS, CrGS and CrG10H was carried out by adding 0.1 mg of each purified protein (microsomal pellet in case of CrG10H) to an assay mixture containing IPP (0.1 mM), DMAPP (0.1 mM), NADPH (1 mM), Glucose 6-phosphate (2.5 μM), Glucose 6-phosphate dehydrogenase (1U), FAD (10 μM), FMN (10 μM) in buffer (100 mM K_2_HPO_4_, 50 mM MOPSO, pH 7.6, 1 mM EDTA, 1 mM DTT, 10 mM MgCl_2_, 0.1 mM MnCl_2_) and incubated on a metabolic shaker for 3 hours at 50 rpm. After this incubation period, the aqueous phase was extracted three times with 0.5 mL of dichloromethane. The combined organic phase was dried over sodium sulphate, concentrated and subjected to GC and GC-MS analyses.

The Cr10HGO and CrIDS combined assay was carried out by adding 0.1 mg of each purified protein to an assay mixture containing 0.2 mM 10-hydroxygeraniol and 0.2 mM of NADP^+^ in assay buffer (20 mM MOPS, pH 7.0, 10% v/v Glycerol) and incubated at 30°C on a rotary shaker. Incubations were carried out for different time intervals (30 min to 6 hours). After this incubation period, the assay samples were exctrated thrice with 0.5 mL dichloromethane. Similarly, the combined assay for CrG10H, Cr10HGO and CrIDS was carried out in 2 mL assay buffer (100 mM K_2_HPO_4_, 50 mM MOPS, pH 7.6, 1 mM EDTA, 1 mM DTT, 10 mM MgCl_2_, 0.1 mM MnCl_2_) containing the required cofactors and geraniol as substrate.

### Product Analysis

1 μl of the extract was injected onto a i) 30 m × 0.25 mm × 0.25 μm HP-5 capillary GC column with a temperature gradient from 60 to 120°C at 20°C per min, followed by a temperature gradient from 120 to 170°C at 2.5°C per min and a final temperature gradient from 170 to 190°C at 20°C per min (program 1) or ii) 30 m × 0.25 mm × 0.12 μm Astec CHIRAL DEX™ B-DA capillary column with a temperature gradient from 60 to 100°C at 4°C per min, followed by a temperature gradient from 60 to 160°C at 1°C per min, followed by a temperature gradient from 160 to 215°C at 10°C per min (program 2) or iii) 30 m × 0.25 mm × 0.12 μm Astec CHIRAL DEX™ B-DA Capillary Column with a temperature gradient from 60 to 160°C at 1°C per min, followed by a temperature gradient from 160 to 215°C at 10°C per min (program 3). Nitrogen was used as a carrier gas at a flow rate of 1 mL/min. Analyses by GC-MS were carried out under similar conditions at a helium flow rate of 1 mL/min.

^1^H and ^13^C NMR in CDCl_3_ spectra were recorded at 400.13 and 100.63 MHz. Chemical shifts are given in *δ*-values relative to TMS (tetramethylsilane) as internal standard. Exact molecular mass and molecular formula determinations were recorded using Q Exactive Orbitrap spectrometer.

## Author Contributions

Isolation of genes and cloning was done by R.K. Protein purification was done by R.K., P.L.S., B.R. and S.M. Synthesis of various compounds was carried out by S.P.K. and M.K.C. R.K. and B.R. carried out the enzyme assays and kinetics. H.V.T. has conceptualized, supervised and acted as overall study director.

## Supplementary Material

Supplementary Information Supplementary Information

## Figures and Tables

**Figure 1:**
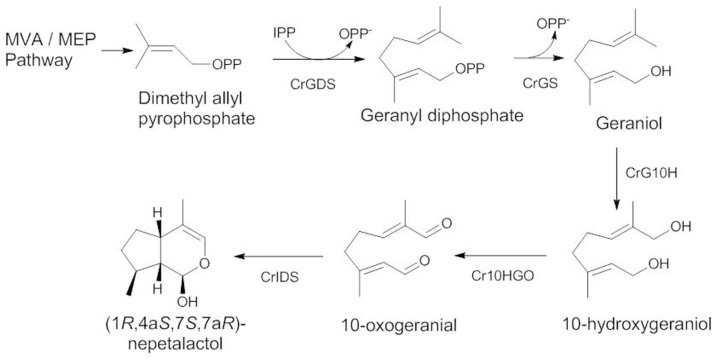
Iridoid Biosynthetic Pathway cyclizes 10-oxogeranial into equilibrium mixture of cis-trans-nepetalactol and iridodials. The bicyclic compound, cis-trans-nepetalactol is the key intermediate.

**Table 1:** Steady state kinetics of Cr10HGO

Substrate	K_m_ (μM)	V_max_ (μM/sec^−1^)	V_max_/E_t_ (sec^−1^)
10-hydroxygeraniol	1.50	0.019	7.25
NADP^+^	1.34	0.020	7.75
NAD+	0.39	0.008	3.29
10-hydroxygeranial	1.10	0.016	6.10
10- oxogeraniol	1.40	0.013	5.05
NADPH	1.07	0.014	5.65
10-oxogeranial	1.32	0.014	5.44
10-hydroxynerol	1.35	0.013	4.90
