# Supplementary material for: Characterization of 10-Hydroxygeraniol Dehydrogenase from Catharanthus roseus Reveals Cascaded Enzymatic Activity in Iridoid Biosynthesis
Source: Sci Rep. 2015 Feb 5;5:8258. doi: 10.1038/srep08258 (PMC4317706; doi:10.1038/srep08258)
Supplement: Supplementary Information [file srep08258-s1.doc]

Supplementary Information

Characterization of 10-hydroxygeraniol Dehydrogenase from *Catharanthus roseus* Reveals Cascaded Enzymatic Activity in Iridoid Biosynthesis

Ramakrishnan Krithika, Prabhakar Lal Srivastava, Bajaj Rani, Swati P. Kolet, Manojkumar Chopade, Mantri Soniya, and Hirekodathakallu V. Thulasiram*****

**Supplemental Tables**

**Table S1:** Transcripts from Transcriptome Analysis of Vinca

| **Transcript ID** | **Length of ORF** | **BLAST Results** | **% Similarity** |
| --- | --- | --- | --- |
| **MEP Pathway Transcripts** | | | |
| Vinca_1853 | 2151 | *Catharanthus roseus* mRNA for 1-deoxyxylulose 5-phosphate synthase | 99% |
| Vinca_1617 | 1425 | *Catharanthus roseus* 1-deoxy-D-xylulose-5-phosphate reductoisomerase (dxr) mRNA, complete cds | 99% |
| Vinca_9009 | 936 | *Catharanthus roseus* 4-diphosphocytidyl-methylerythritol 2-phosphate synthase mRNA, complete cds | 99% |
| Vinca_2073 | 1227 | *Catharanthus roseus* 4-diphosphocytidyl-2-C-methyl-D-erythritol kinase (CMK) mRNA, complete cds | 99% |
| Vinca_2177 | 711 | *Catharanthus roseus* 2C-methyl-D-erythritol 2,4-cyclodiphosphate synthase (MECS) mRNA, complete cds | 98% |
| Vinca_481 | 2223 | *Catharanthus roseus* GCPE protein mRNA, complete cds | 99% |
| Vinca_465 | 1389 | *Catharanthus roseus* 1-hydroxy-2-methyl-butenyl 4-diphosphate reductase mRNA, complete cds | 99% |
| Vinca_775 | 939 | *Catharanthus roseus* plastid isopentenyl pyrophosphate:dimethylallyl pyrophosphate isomerase isoform 1 mRNA, complete cds; nuclear gene for plastid product | 99% |
| **MVA Pathway Transcripts** | | | |
| Vinca_1158 | 1218 | *Catharanthus roseus* acetoacetyl-CoA thiolase mRNA, complete cds | 98% |
| Vinca_2418 | 1392 | *Catharanthus roseus* hydroxymethylglutaryl-CoA synthase mRNA, complete cds | 99% |
| Vinca_1892 | 1614 (3’ and 5’ ends missing) | *Camellia sinensis* cultivar Longjing 43 HMG-CoA reductase (HMGR1) mRNA, complete cds | 79% |
| Vinca_4366 | 1163 | *Catharanthus roseus* mevalonate kinase mRNA, complete cds | 99% |
| Vinca_9276 | 1497 | *Catharanthus roseus* 5-phosphomevalonate kinase mRNA, complete cds | 99% |
| Vinca_8419 | 1265 | *Catharanthus roseus* mevalonate 5-diphosphate decarboxylase mRNA, complete cds | 99% |
| Vinca_775 | 939 | *Catharanthus roseus* plastid isopentenyl pyrophosphate:dimethylallyl pyrophosphate isomerase isoform 1 mRNA, complete cds; nuclear gene for plastid product | 99% |
| **Monoterpene Iridoid Pathway Transcripts** | | | **NCBI GenBank Accession Numbers** |
| Vinca_5406 | 1263 | Geranyl diphosphate synthase (CrGDS) | KF561462 |
| Vinca_2171, Vinca_3238 | 1770 | Geraniol synthase (CrGS) | KF561459 |
| Vinca_742 | 1482 | Geraniol 10-hydroxylase (CrG10H) | KF561461 |
| Vinca_536, Vinca_18357 | 1083 | 10-hydroxygeraniol dehydrogenase (Cr10HGO) | KF561458 |
| Vinca_2280 | 1161 | Iridoid synthase (CrIDS) | KF561460 |

**Table S2:** Product ratio studies of Cr10HGO with various acyclic compounds

| **S. No.** | **Substrate** | **% Product Formed*** |
| --- | --- | --- |
| 1 | Lavandulol | 0 |
| 2 | Geranygeraniol | 0 |
| 3 | β - Citronellol | 0 |
| 4 | Linalool | 0 |
| 5 | Geranyl acetate | 0 |
| 6 | Linalyl acetate | 0 |
| 7 | Farnesol | 8 |
| 8 | Geraniol | 17 |
| 9 | 10-hydroxygeraniol | 75.2 |
| 10 | 10-hydroxygeranial | 59 |
| 11 | 10-oxogeraniol | 62.3 |
| 12 | 10-oxogeranial | 73.5 |
| 13 | Nerol | 7.5 |
| 14 | Trans- Nerolidol | 0 |
| 15 | α - Bisabolol | 0 |
| 16 | Eugenol | 0 |
| 17 | Dihydromyrcenol | 0 |
| 18 | Menthol | 0 |
| 19 | Chrysanthemyl alcohol | 0 |
| 20 | α - Santalol | 0 |
| 21 | Geranyllinalool | 0 |

*Rest of the % is unreacted substrate

**Table S3:** Strains and plasmids used in this study.

| **Strains / Plasmids** | **Characteristics** | **Sources** |
| --- | --- | --- |
| **Strains** |  |  |
| *E.coli* |  |  |
| TOP 10 | Host strain for cloning | Invitrogen |
| BL21 (DE3) | Host strain for expression | Novagen |
| Rosetta2 (DE3) | Host strain for expression | Novagen |
| *S.cerevisiae* |  |  |
| INVSc1 | Host strain for yeast expression | Invitrogen |
| **Plasmids** |  |  |
| Zero Blunt | Kan^r^, cloning vector | Invitrogen |
| pRSETb | Amp^r^, expression vector | Invitrogen |
| pET32a | Amp^r^, expression vector | Novagen |
| pET28a | Kan^r^, expression vector | Novagen |
| pYES2 | Yeast expression vector | Invitrogen |

**Table S4: Cloning strategies for Cr10HGO, CrGDS, CrGS, CrG10H and CrIDS.**

| **Gene** | **Oligonucleotide Primer Sequences** |
| --- | --- |
| Cr10HGO | Fwd Primer: 5'- ATGGCGAAATCACCGGAAGTCGAGC-3’  Rev Primer: 5'-TTATGCAGATTTCAGTGTGTTGGCT-3' |
| CrGDS | Fwd Primer: 5'- ATGTTGTTTTCCAGAGGATTGTATA -3’  Rev Primer: 5'- TCACTTTCTTCTTGTAATAACGCGT -3' |
| CrGS | Fwd Primer: 5'- ATGGCAGCCACAATTAGTAACCTTT-3’  Rev Primer: 5'-TTAAAAACAAGGTGTAAAAAACAAAGC -3' |
| CrG10H | Forward Primer: 5'- ATGGATTACCTTACCATAATATTAA -3’ Reverse Primer: 5'- TTAAAGGGTGCTTGGTACAGCACGC -3' |
| CrIDS | Fwd Primer: 5'-ATGAGTTGGTGGTGGAAGAGGTCCA-3’  Rev Primer: 5'-CTAAGGAATAAACCTATAATCCCTC-3' |

**Table S5: Bacterial Expression**

| **Gene** | **Expression Vector** | **Host strain for expression** | **Induction conditions** |
| --- | --- | --- | --- |
| Cr10HGO | pRSETb | BL21 (DE3) | 1 mM IPTG, 30 ᵒC for 6 hours |
| CrGDS | pET28a | Rosetta2 (DE3) | 0.5 mM IPTG, 16 ᵒC for 18 hours |
| CrGS | pET32a | Rosetta2 (DE3) | 1 mM IPTG, 16ᵒC for 12 hours |
| CrIDS | pET32a | Rosetta2 (DE3) | 1 mM IPTG, 16ᵒC for 12 hours |

**Supplemental Figures**


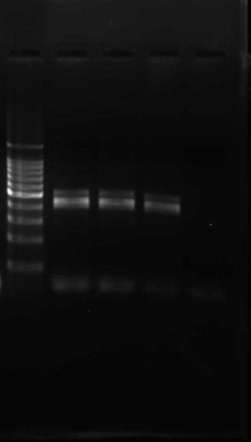


**1 2 3 4 5**

**Legend**

**Lane 1 :** DNA Ladder

**Lane 2 :** PCR product of Cr10HGO from White Stem cDNA

**Lane 2 :** PCR product of Cr10HGO from White Leaf cDNA

**Lane 2 :** PCR product of Cr10HGO from White Root cDNA

**Lane 5 :** Negative Control

**Figure S1:** PCR amplification of 10-hydroxygeraniol dehydrogenase from cDNA


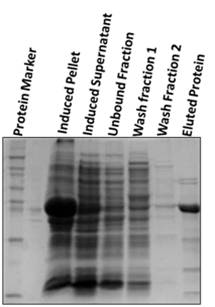


**Figure S2:** SDS-PAGE of protein purification of 10-hydroxygeraniol dehydrogenase


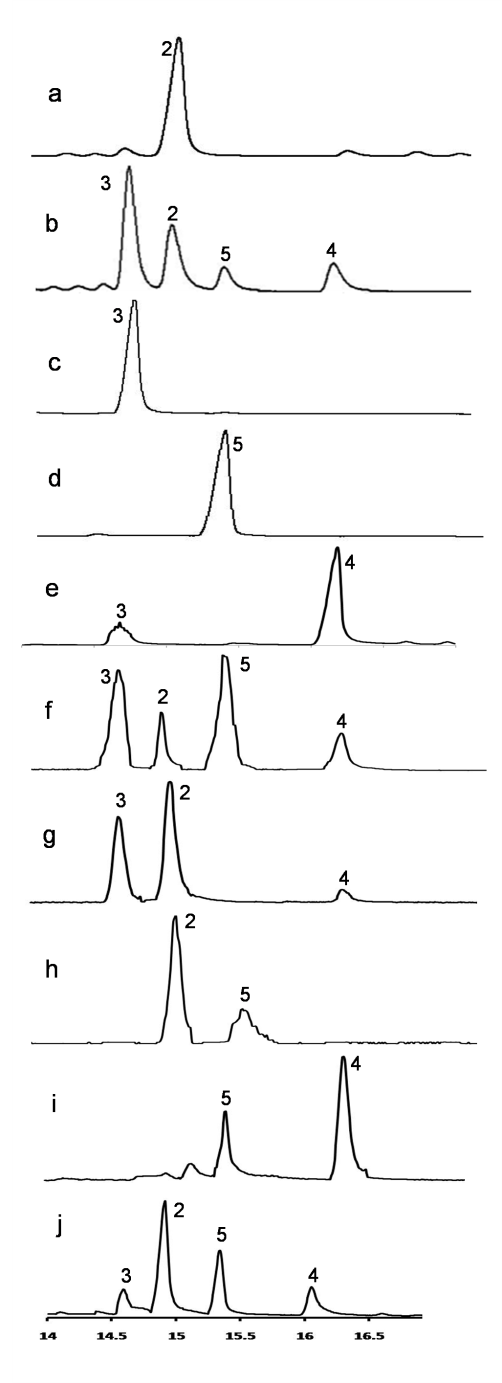


**Figure S3.** Total ion chromatograms (TICs) for 10-hydroxygeraniol dehydrogenase (Cr10HGO) catalyzed reactionswith (a) 10-hydroxygeraniol substrate control, (b) 10-hydroxygeraniol and NADP^+^, (c) 10-oxogeraniol standard, (d) 10-oxogeranial standard, (e) 10-hydroxygeranial standard, (f) 10-oxogeraniol and NADP^+^, (g) 10-oxogeraniol and NADPH, (h) 10-oxogeranial and NADPH, (i) 10-hydroxygeranial and NADP^+^, (j) 10-hydroxygeranial and NADPH. The peaks represent (**2**) 10-hydroxygeraniol, (**3**) 10-oxogeraniol, (**4**) 10-hydroxygeranial, and (**5**) 10-oxogeranial.

**
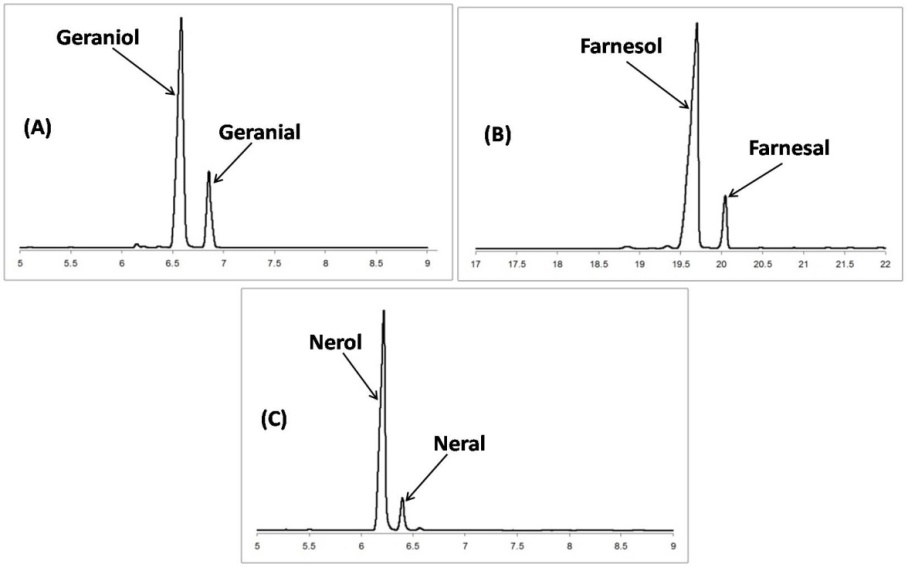
**

**Figure S4:** TICs of reaction of Cr10HGO with Geraniol (A), Farnesol (B) and Nerol (C) showing formation of geranial, farnesal and neral, respectively


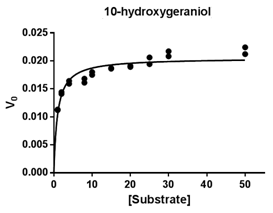


**Figure S5:** Michaelis-Menten Plot for 10-hydroxygeraniol kinetics

**
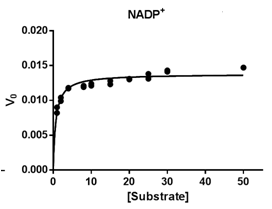
**

**Figure S6:** Michaelis-Menten Plot for NADP^+^kinetics

**
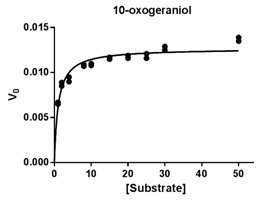
**

**Figure S7:** Michaelis-Menten Plot for 10-oxogeraniolkinetics

**
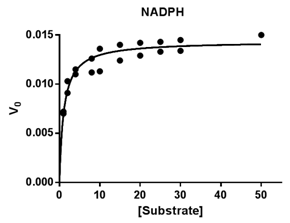
**

**Figure S8:** Michaelis-Menten Plot for NADPH kinetics

**
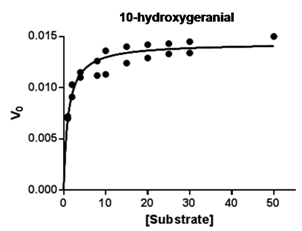
**

**Figure S9:** Michaelis-Menten Plot for 10-hydroxygeranial kinetics

**
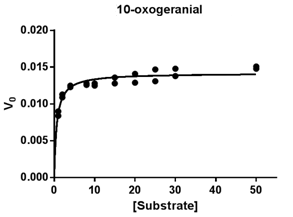
**

**Figure S10:** Michaelis-Menten Plot for 10-oxogeranial kinetics

**
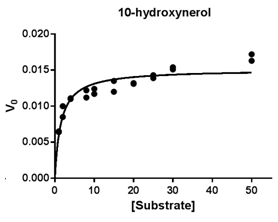
**

**Figure S11:** Michaelis-Menten Plot for 10-hydroxynerol kinetics

**
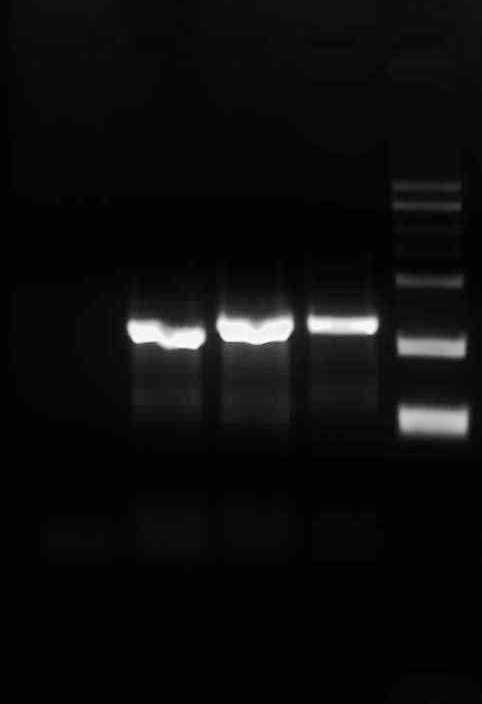
**

**1 2 3 4 5**

**Legend**

**Lane 1 :** Negative Control

**Lane 2 :** PCR product of CrIDS from White Stem cDNA

**Lane 2 :** PCR product of CrIDS from White Leaf cDNA

**Lane 2 :** PCR product of CrIDS from White Root cDNA

**Lane 5 :** DNA Ladder

**Figure S12:** PCR amplification of Iridoid synthase from cDNA


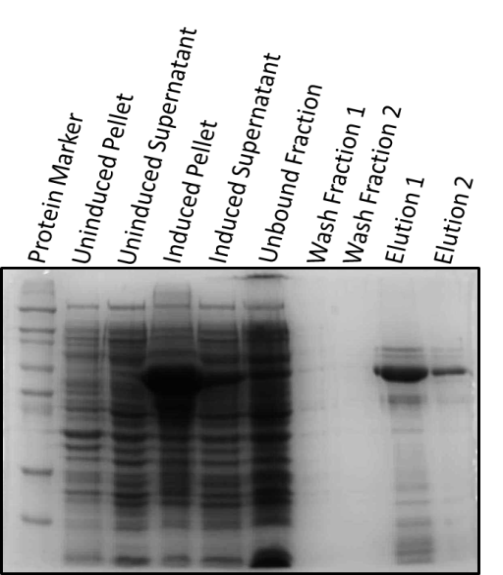


**Figure S13:** SDS-PAGE of protein purification of Iridoid synthase


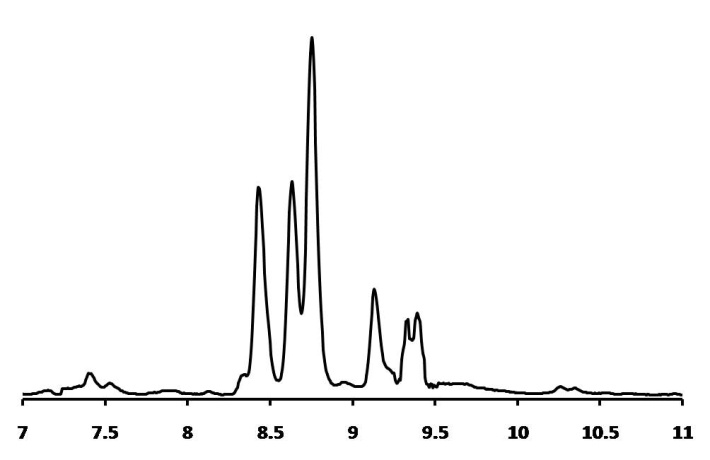


**Figure S14:** TIC of assay extract of CrIDS. 0.1 mg of protein in 0.5 mL MOPS buffer (20 mM MOPS, pH 7.0, 10% v/v Glycerol), in the presence of 200 µM NADPH at 30 °C for 30 minutes with 0.2 mM of 10-oxogeranial.

**
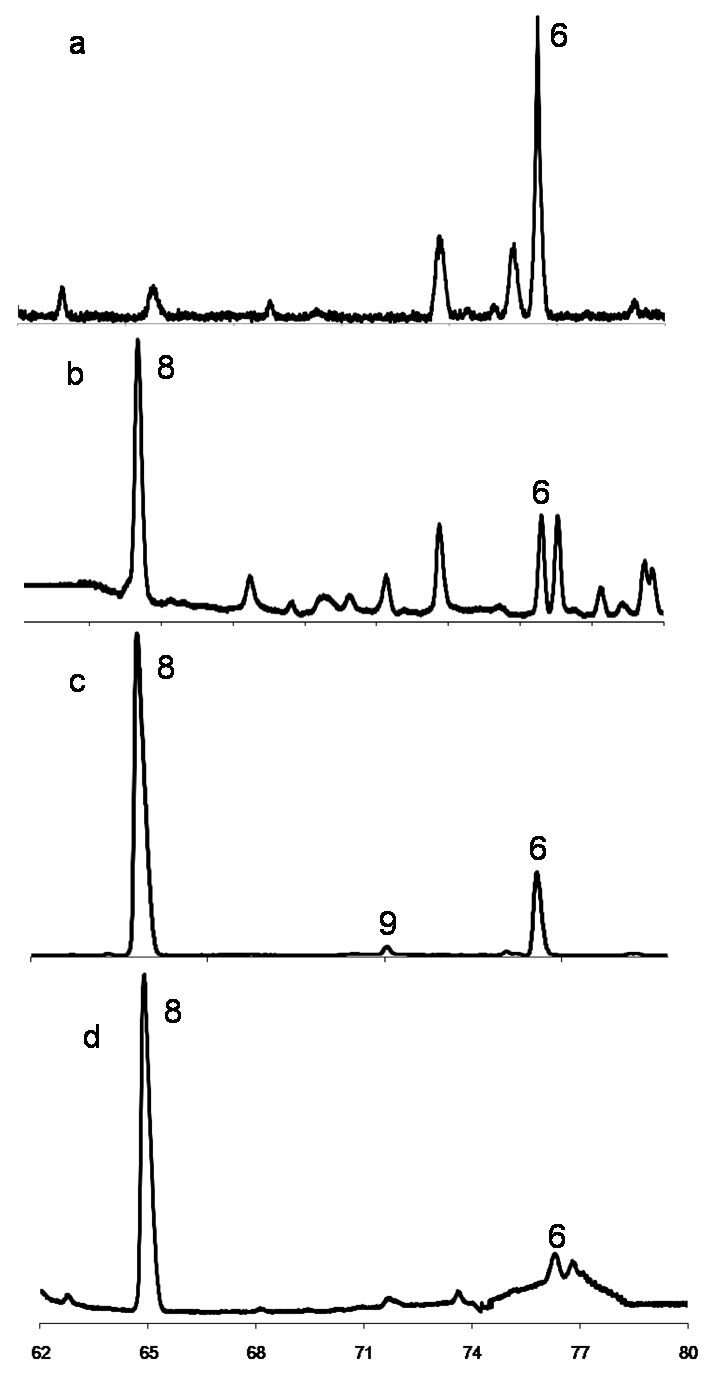
**

**Figure S15:** Comparison of TICs of (**a**) Cr10HGO+CrIDS assay, (**b**) Acetylated Cr10HGO+CrIDS assay mixture, (**c**) Acetylated mixture of synthesized *cis-trans* nepetalactol and (**d**) Co-injection of acetylated mixture of synthesized *cis-trans* nepetalactol and acetylated Cr10HGO+CrIDS assay mixture showing the peaks, *cis-trans*-nepetalactol(**6**), acetylated (1*R*, 4a*S*, 7*S*, 7a*R*)-nepetalactol (**8**), acetylated (1*S*, 4a*S*, 7*S*, 7a*R*)-nepetalactol (**9**).

In chromatogram (**b**), there is a small peak, which seems to be in the same position as **9**. However, mass fragmentation analyses indicated that this is not corresponds to acetylated nepetalactol (**9**) as observed M^+^ is 152 instead of 210 ( Supplementary Fig. S16 and S17).

**
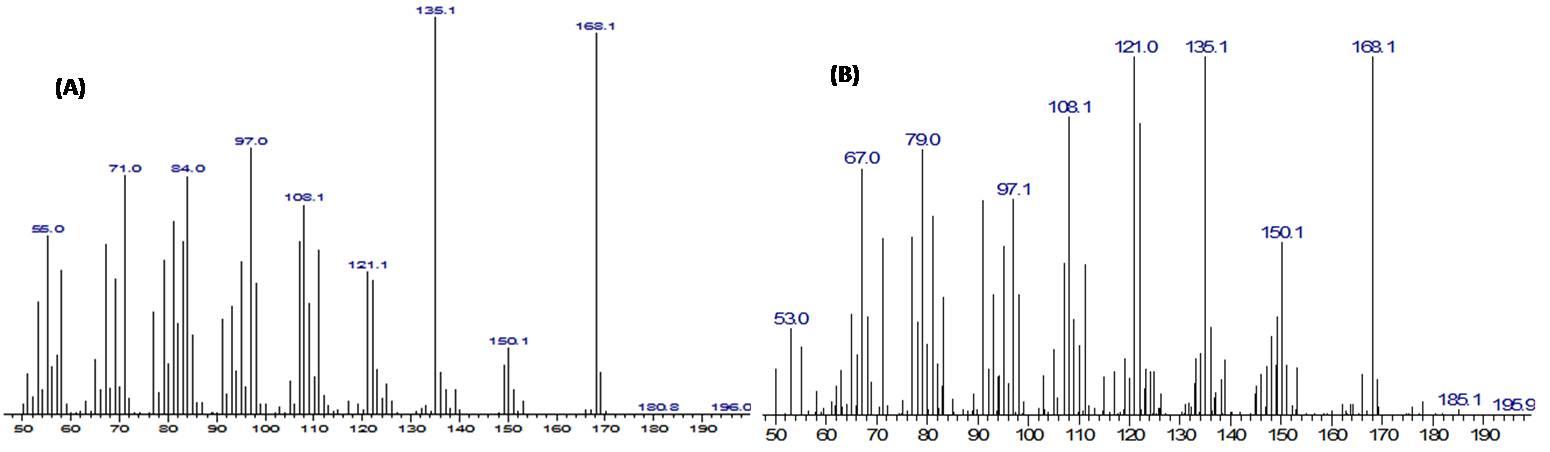
**

**Figure S16:** EI MS of: (A) nepetalactol from Cr10HGO+CrIDS assay and (B) synthesised nepetalactol (**6)**


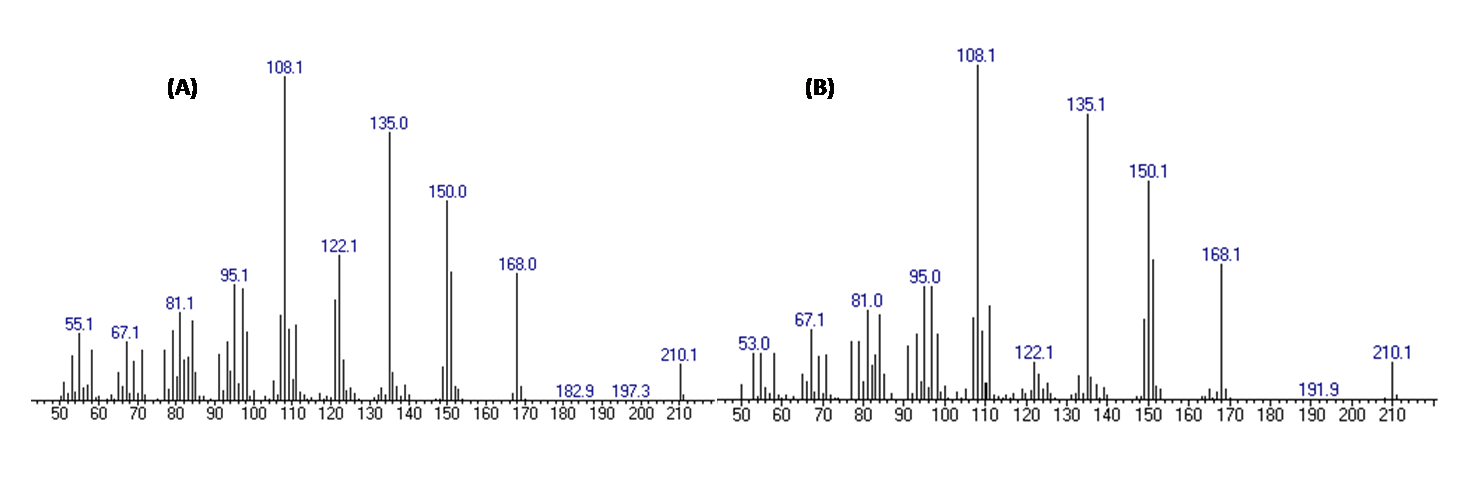


**Figure S17:** EI MS of (A) acetylated (1*R*, 4a*S*, 7*S*, 7a*R*)-nepetalactol from acetylation of synthesized nepetalactol (**8**) and (B) (1*R*, 4a*S*, 7*S*, 7a*R*)-nepetalactol from acetylation of Cr10HGO+CrIDS assay.

**
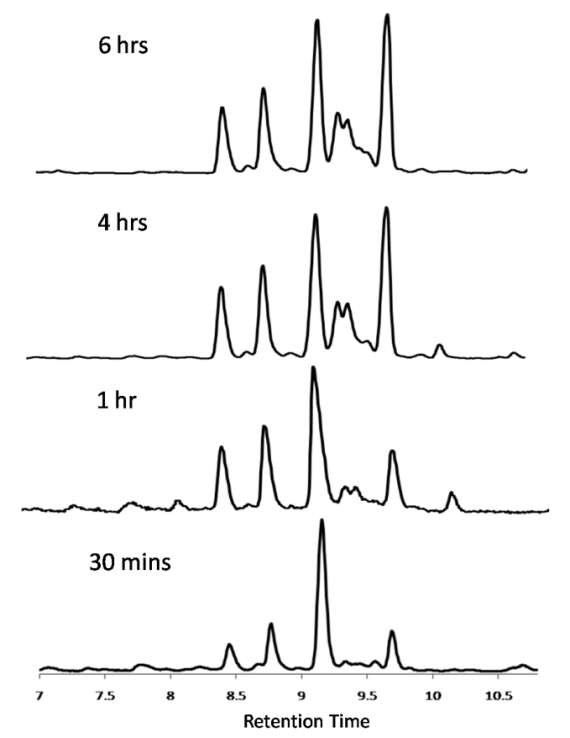
**

**Figure S18:** TICs of the CH_2_Cl_2_ extracts of the time course of the enzymatic reactions of

Cr10HGO and CrIDS on 10-hydroxygeraniol and NADP^+^ as substrates.

**
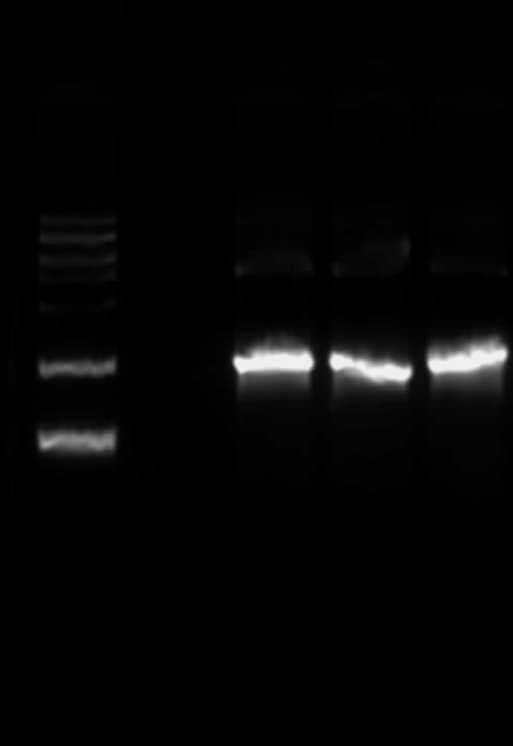
**

**1 2 3 4 5**

**Legend**

**Lane 1 :** DNA Ladder

**Lane 2 :** Negative Control

**Lane 3 :** PCR product of CrGDS from White Root cDNA

**Lane 4 :** PCR product of CrGDS from White Leaf cDNA

**Lane 5 :** PCR product of CrGDS from White Stem cDNA

**Figure S19:** PCR amplification of Geranyldiphosphate synthase from cDNA

**1 2 3 4 5**

**
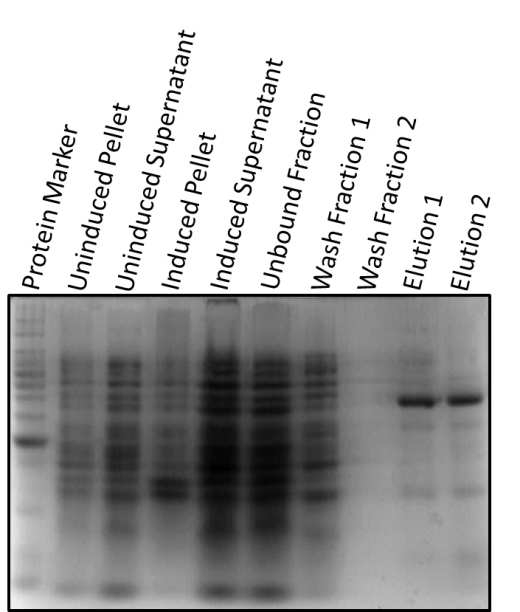
**

**Figure S20:** SDS-PAGE of protein purification of Geranyl diphosphate synthase


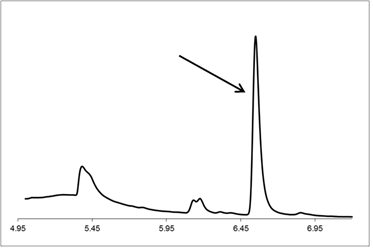


**Geraniol**

**Figure S21:**TIC of assay extract of CrGDS. 0.2 mg of protein in 0.5 mL of MOPSObuffer (50 mM MOPSO, pH 7.0, 10% v/v Glycerol, 2 mM DTT, 10 mM MgCl_2_), incubated at 30 °C for 6 hourswith 0.2 mM each of IPP and DMAPP. Further incubation at 30 °C, overnight, with 5 U Alkaline phosphatase to hydrolyze the GPP formed, so that it may be detected by GC / GC-MS.

**
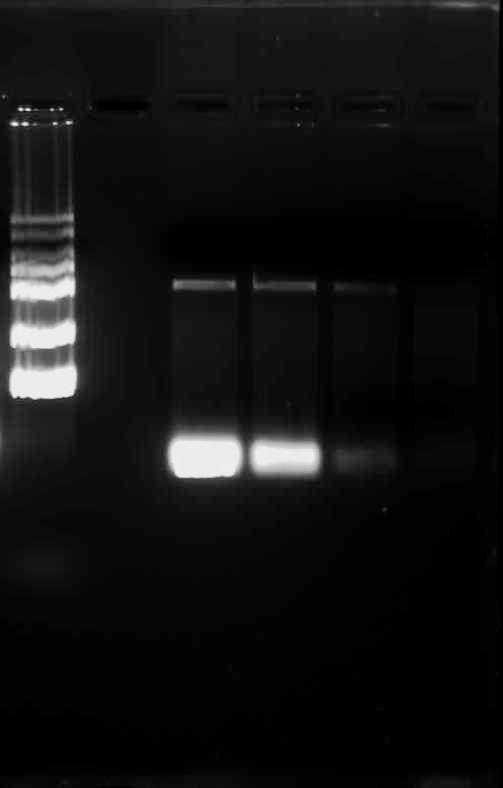
**

**1 2 3 4 5**

**Legend**

**Lane 1 :** DNA Ladder

**Lane 2 :** PCR product of CrGS from White Stem cDNA

**Lane 2 :** PCR product of CrGS from White Leaf cDNA

**Lane 2 :** PCR product of CrGS from White Root cDNA

**Lane 5 :** Negative Control

**Figure S22:** PCR amplificationof Geraniol synthase from cDNA


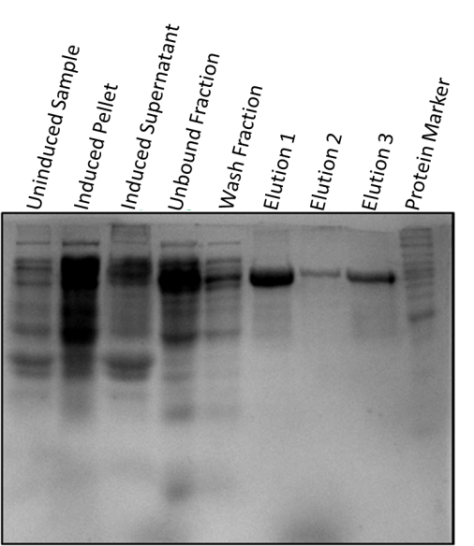


**Figure S23:** SDS-PAGE of protein purification of Geraniol synthase


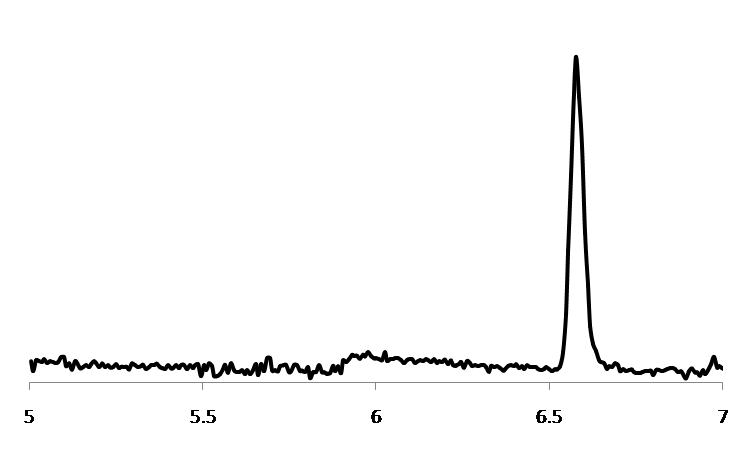


**Geraniol**

**Figure S24:** TIC of assay extract of CrGS. 0.1 mg of protein in 0.5 mL HEPES buffer (100mM HEPES-KOH, pH 7.0, 1 mM MgCl_2_, 100µM MnCl_2_, 10% v/v Glycerol) incubated at 30 °C for 1 hour with 0.2 mM of GPP.

**
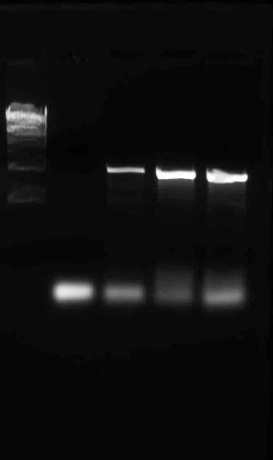
**

**1 2 3 4 5**

**Legend**

**Lane 1 :** DNA Ladder

**Lane 2 :** Negative Control

**Lane 3 :** PCR product of CrG10H from White Root cDNA

**Lane 4 :** PCR product of CrG10H from White Leaf cDNA

**Lane 5 :** PCR product of CrG10H from White Stem cDNA

**Figure S25:** PCR amplificationof Geraniol 10-hydroxylase from cDNA

**
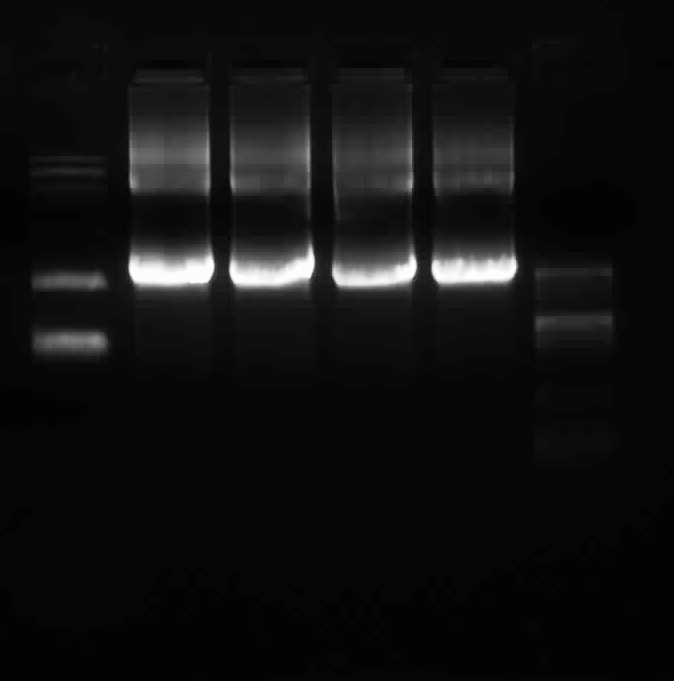
**

**1 2 3 4 5**

**Legend**

**Lane 1 :** DNA Ladder

**Lanes 2-5 :** CrG10H Clones 1-4

**Figure S26:** Colony PCR amplificationof Geraniol 10-hydroxylase Clones in pYES2


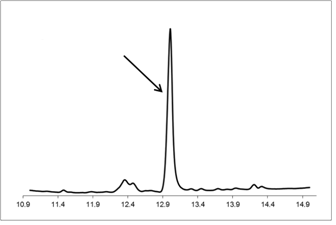


**10-Hydroxygeraniol**

**Figure S27:** TIC of assay extract of CrG10H. 1 mg of microsomal fraction in 1 ml potassium phosphatebuffer (0.1 M K_2_HPO_4_, pH 7.6, 1 mM EDTA, 1 mM DTT), in the presence of 1 mM NADPH, 2.5 µM Glucose 6-phosphate, 1U Glucose 6-phosphate dehydrogenase, 10 µM FAD, 10 µM FMN, with the addition of 0.5 mM Geraniol was incubated at 30 °C for 1 hour.

**
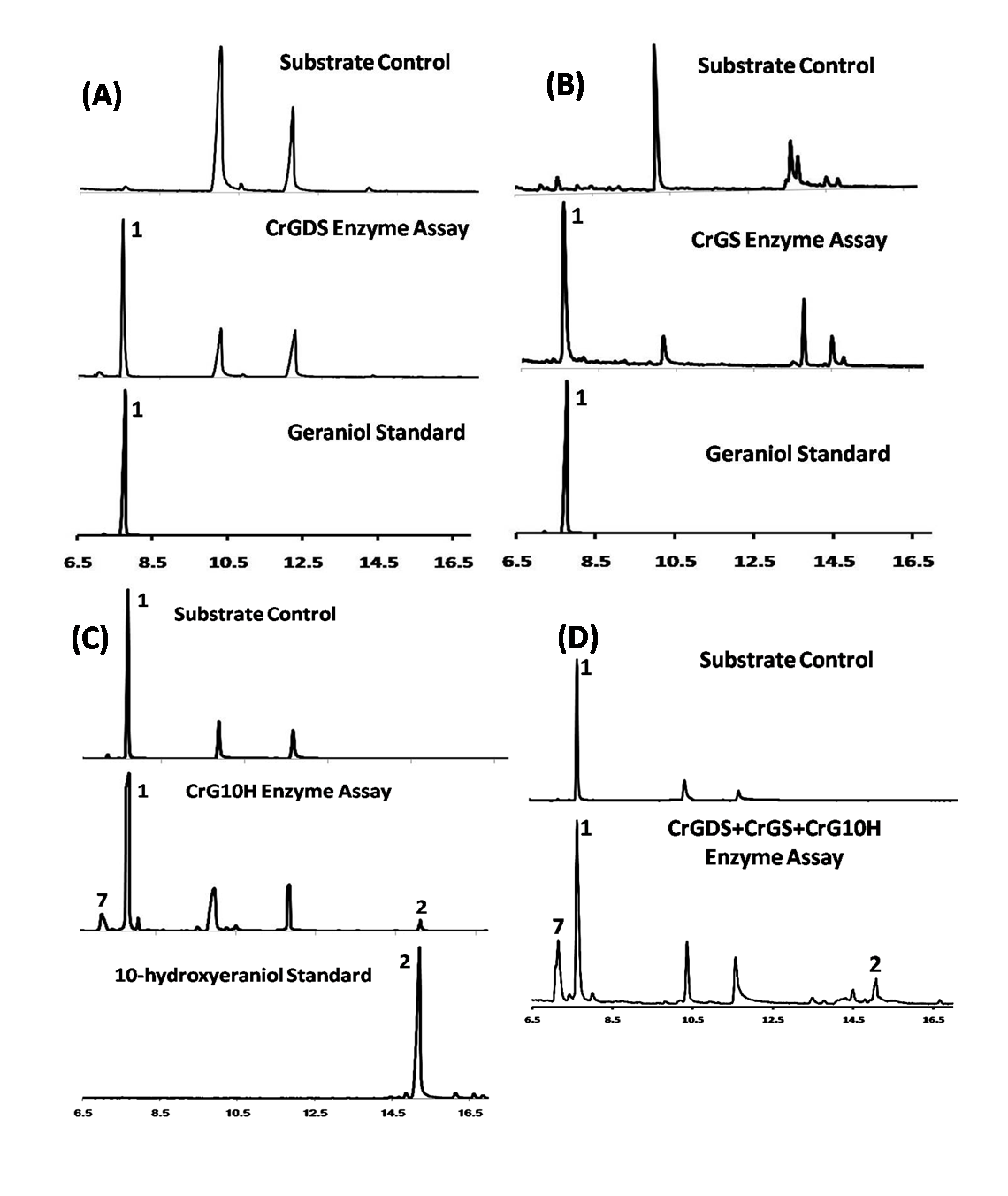
**

**Figure S28:** (A) TIC of CrGDS assay in comparison with the control and standard, (B) TIC of CrGS assay in comparison with the control and standard, (C) TIC of CrG10H assay in comparison with the control and standard, (D) TIC of CrGDS+CrGS+CrG10H assay in comparison with the control and standard; (**1**)-Geraniol, (**2**)- 10-hydroxygeraniol, (**7**)-Nerol; Substrate Control - buffer+substrate+co-factor (wherever applicable), Enzyme Assay - buffer+substrate+co-factor (wherever applicable)+enzyme. The unlabelled peaks were observed in controls also.

**
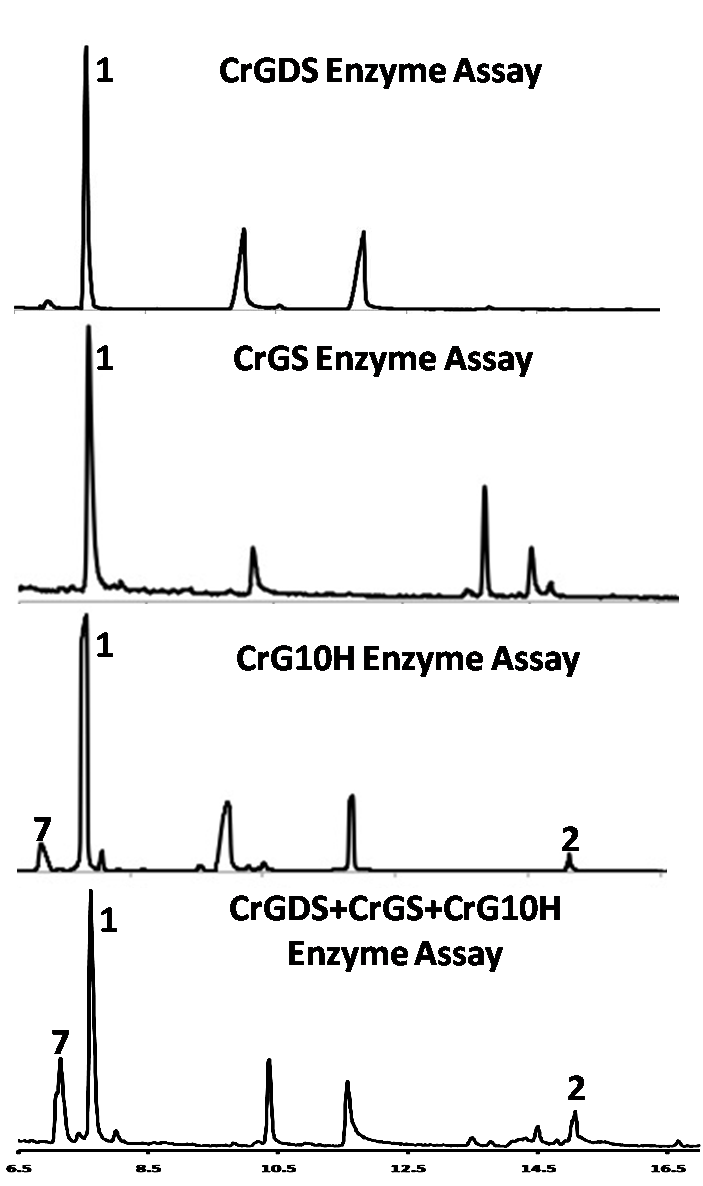
**

**Figure S29:** Comparison of TICs of CrGDS, CrGS, CrG10H and CrGDS+CrGS+CrG10H assays; 1-Geraniol, 2- 10-hydroxygeraniol, 3-Nerol; all unlabelled peaks were observed in controls also; (**1**)-Geraniol, (**2**)- 10-hydroxygeraniol, (**7**)-nerol.

**
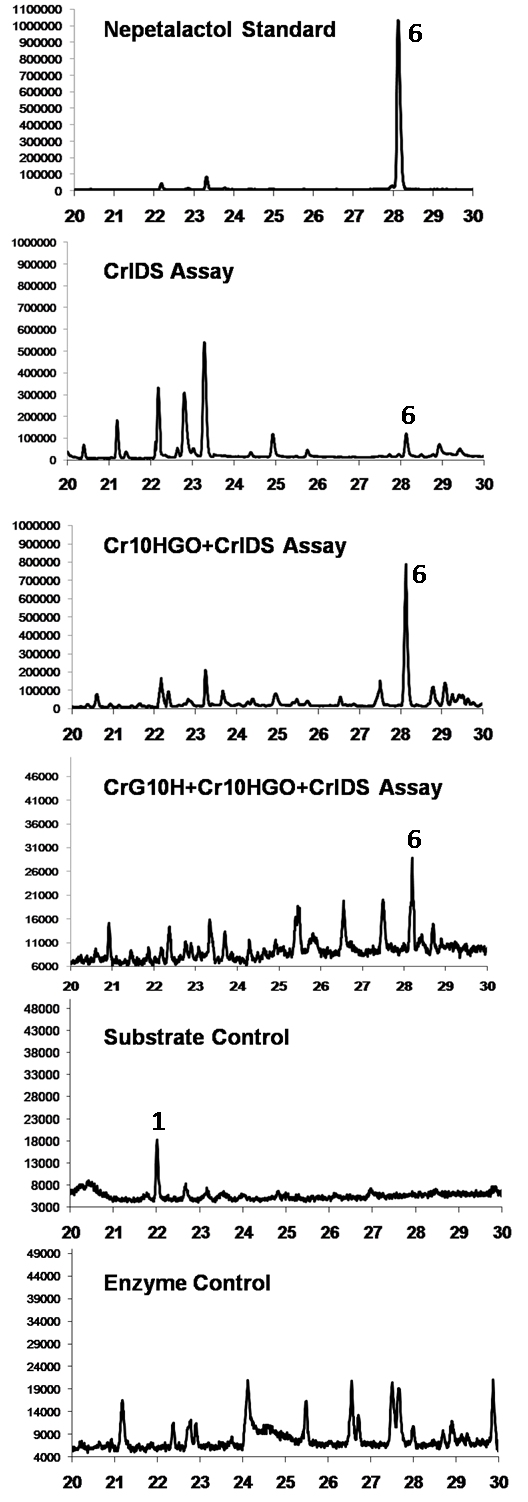
**

**Figure S30:** Comparison of TICs of CrIDS, Cr10HGO+CrIDS and CrG10H+Cr10HGO+CrIDS assays with substrate control (buffer + Geraniol) and enzyme control (buffer+CrG10H+Cr10HGO+CrIDS); (**1**)- geraniol, (**6**)-(1*R*, 4a*S*, 7*S*, 7a*R*)-nepetalactol, on Astec CHIRALDEX^TM^ B-DA column with a temperature gradient from 60 to 140 °C at 4 °C per min, followed by a temperature gradient from 140 to 190 °C at 2.5 °C per min with a He flow rate of 1 mL/ min.


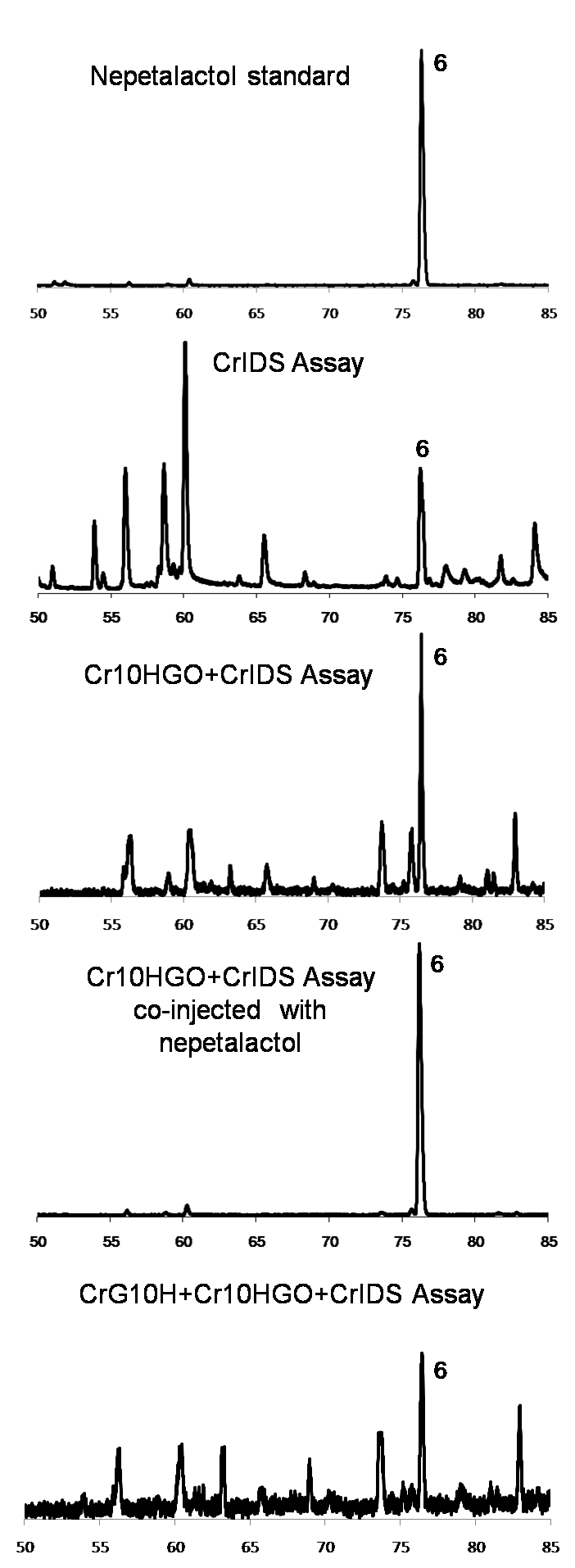


**Figure S31:** Comparison of TICs of CrIDS, Cr10HGO+CrIDS and CrG10H+Cr10HGO+CrIDS assays with standard (1*R*, 4a*S*, 7*S*, 7a*R*)-nepetalactol on Astec CHIRALDEX^TM^ B-DA column with a temperature gradient from 60 to 160 °C at 1 °C per min, followed by a temperature gradient from 160 to 220 °C at 10 °C per min with a He flow rate of 1 mL/ min.


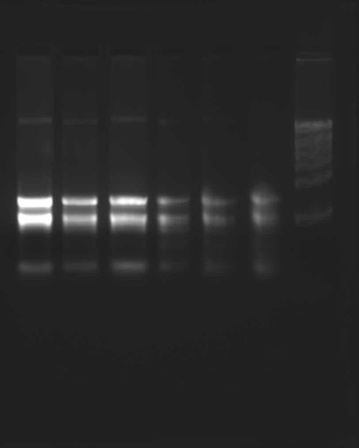


**Legend**

**Lane 1** : RNA from White Leaves

**Lane 2** : RNA from White Stem

**Lane 3** : RNA from White Roots

**Lane 4** : RNA from Pink Leaves

**Lane 5** : RNA from Pink Stem

**Lane 6** : RNA from Pink Roots

**Lane 7** : DNA Ladder

**1 2 3 4 5 6 7**

**Figure S32:** RNA from different tissues of *Catharanthus roseus*


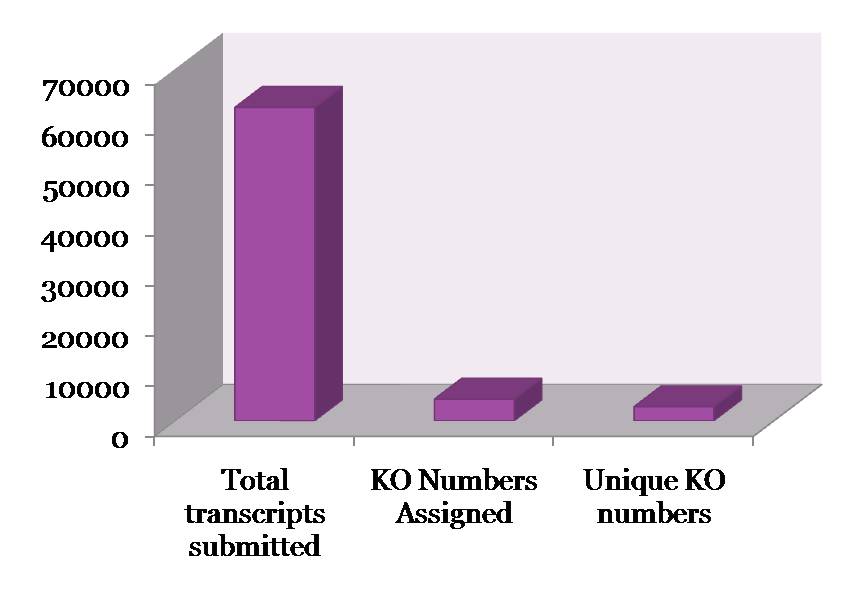


**Figure S33:** Graph representing the number of transcripts assigned unique KO numbers.

This study identifies the probable number of unique transcripts, eliminating

the multiple copy numbers.

**
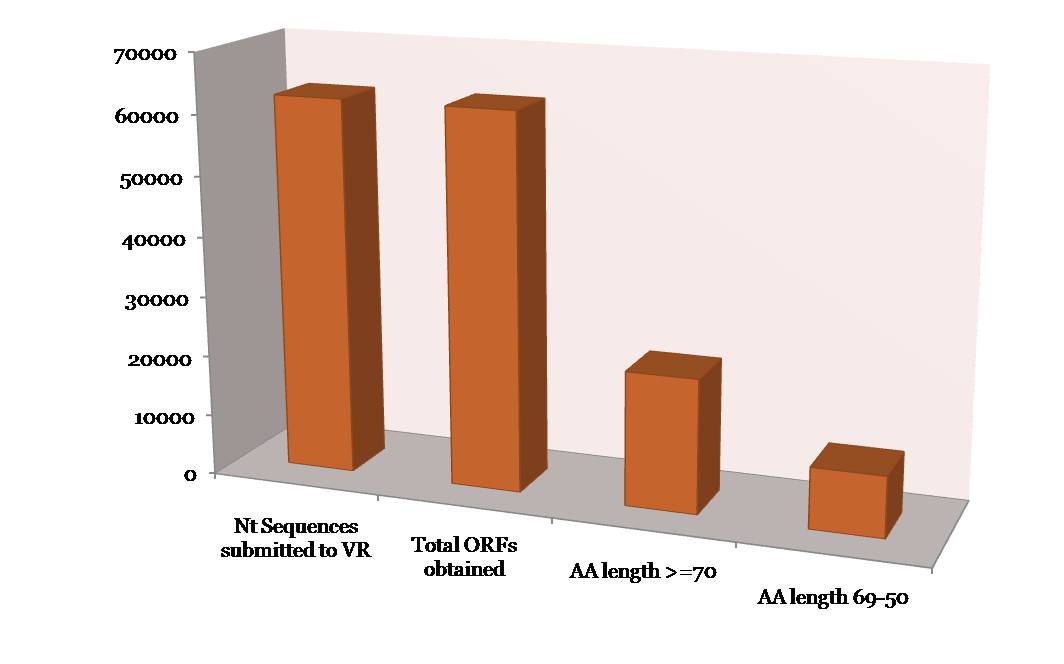
**

**Figure S34:** Graph representing the number of ORFs obtained (after translation to amino acid sequences) when the nucleotide sequences were submitted to Virtual Ribosome. These ORFs were used in further annotational studies

**Scheme S1:** Synthesis of 10-hydroxygeraniol (**2**), 10-oxogeraniol (**3**), 10-hydroxygeranial (**4**), 10-oxogeranial (**5**)and 10- hydroxynerol (**12**)

**Scheme S2:** Synthesis of (1*R*, 4a*S*, 7*S*, 7a*R*)-nepetalactol (**6**) and acetylated (1*R*, 4a*S*, 7*S*, 7a*R*)-nepetalactol (**8**)

**Spectral data**

**10-hydroxygeraniol (2):**

60% yield, colourless liquid, **^1^H NMR** (CDCl_3_, 400 MHz, ppm): δ 5.38-5.35 (m, 2H), 4.15 (d, *J* = 6.7 Hz, 2H), 3.97 (s, 2H), 2.15-2.06 (m, 4H), 1.96 (bs, 2H), 1.66 (s, 3H), 1.64 (s, 3H). **^13^C NMR** (CDCl_3_, 100 MHz, ppm): δ 138.5, 135.0, 125.1, 123.8, 68.5, 59.0, 38.9, 25.4, 16.0, 13.6. **HRMS***m/z*: Calculated for C_10_H_18_O_2_Na- 193.1204; found 193.1197 [M+Na]^+^.

**10-oxogeraniol (3):**

72% yield, pale yellow liquid, **^1^H NMR** (CDCl_3_, 400 MHz, ppm):δ 9.38 (s, 1H), 6.50-6.42 (m, 1H), 5.45-5.41 (m, 1H), 4.18 (d, *J* = 6.7 Hz, 2H), 2.54-2.43 (m, 2H), 2.25-2.17 (m, 2H), 1.74 (s, 3H), 1.70 (s, 3H).**^13^C NMR** (CDCl_3_, 100 MHz, ppm): δ 195.2, 153.7, 139.5, 137.8, 124.5, 59.2, 37.7, 27.0, 16.2, 9.2. **HRMS***m/z*: Calculated for C_10_H_16_O_2_Na- 191.1048; found 191.1039 [M+Na]^+^.

**10-hydroxygeranial (4):**

37% yield, yellow liquid, **^1^H NMR** (CDCl_3_, 400 MHz, ppm): δ 9.97 (d, 1H, *J* = 8.0 Hz), 5.88 (d, IH, *J* = 8.0 Hz), 5.34 (m, 1H), 3.97 (s, 2H), 2.25-2.17 (m, 2H), 2.15 (d, 3H, *J* = l.0 Hz), 2.04-1.97 (m, 2H), 1.64 (s, 3H). **^13^C NMR** (CDCl_3_, 100 MHz, ppm): 191.5, 164.0, 135.9, 127.1, 123.3, 67.9, 40.0, 25.0, 17.4, 13.5. **HRMS** *m/z*: Calculated for C_10_H_16_O_2_Na-191.1048; found 191.1039 [M+Na]^+^.

**10-oxogeranial (5):**

88% yield, pale yellow liquid, **^1^H NMR** (CDCl_3_, 400 MHz, ppm): δ 10.02 (d, *J* = 8.1 Hz 1H,), 9.39 (s, 1H), 6.45-6.28 (m, 1H), 5.93 (d, *J* = 6.7 Hz, 1H), 2.60-2.53 (m, 2H), 2.45-2.38 (m, 2H), 2.20 (s, 3H), 1.76 (s, 3H). **^13^C NMR** (CDCl_3_, 100 MHz, ppm): δ194.6, 190.8, 161.3, 151.4, 140.0, 127.5, 38.5, 26.2, 17.4, 9.1. **HRMS***m/z*: Calculated for C_10_H_14_O_2_Na- 189.0891; found 189.0883 [M+Na]^+^.

**(1*R*, 4a*S*, 7*S*, 7a*R*)-nepetalactol (6):**

80% yield, colourless liquid, **^1^H NMR** (CDCl_3_, 400 MHz, ppm): δ 6.00 (s, 1H), 4.83 (m, 1H), 3.28 (s, 1H), 2.44 (m, 1H), 1.97-1.82 (m, 3H) 1.66 (m, 1H), 1.54 (s, 3H), 1.36 (m, 1H), 1.16-1.10 (m, 1H), 1.08 (d, *J* = 6.8 Hz, 3H). **^13^C NMR** (CDCl_3_, 100 MHz, ppm): δ 134.1, 113.7, 94.4, 50.4, 38.7, 35.8, 33.3, 30.8, 20.6, 16.3. **HRMS***m/z*: Calculated for C_10_H_16_O_2_Na- 191.1048; found 191.1041 [M+Na]^+^. **Rotation:** [α] -48.85 (*c* 0.92, CHCl_3_).

**10- hydroxynerol (12):**

69% yield, pale yellow liquid, **^1^H NMR** (CDCl_3_, 400 MHz, ppm): δ 5.46-5.39 (m, 2H), 4.08 (d, *J* = 6.7 Hz, 2H), 3.98 (s, 2H), 2.14 (m, 4H), 1.96 (bs, 2H), 1.75 (s, 3H), 1.65 (s, 3H). **^13^C NMR** (CDCl_3_, 100 MHz, ppm): δ 138.4, 135.6, 124.9, 124.8, 68.4, 58.8, 31.3, 25.2, 23.3, 13.7. **HRMS***m/z*: Calculated for C_10_H_18_O_2_Na- 193.1204; found 193.1197 [M+Na]^+^.

**Figure S35:**^1^H NMR spectrum of **2** in CDCl_3_

**Figure S36:**^13^C NMR spectrum of **2** in CDCl_3_

**Figure S37:** DEPT-135 NMR spectrum of **2** in CDCl_3_

**Figure S38:**^1^H NMR spectrum of **3** in CDCl_3_

**Figure S39:**^13^C NMR spectrum of **3**in CDCl_3_

**Figure S40:** DEPT-135 NMR spectrum of **3** in CDCl_3_

**Figure S41:**^1^H NMR spectrum of **4** in CDCl_3_

**Figure S42:**^13^C NMR spectrum of **4** in CDCl_3_

**Figure S43:** DEPT-135 NMR spectrum of **4** in CDCl_3_

**Figure S44:**^1^H NMR spectrum of **5** in CDCl_3_

**Figure S45:**^13^C NMR spectrum of **5** in CDCl_3_

**Figure S46:** DEPT-135 NMR spectrum of **5**in CDCl_3_

**Figure S47:**^1^H NMR spectrum of **6** in CDCl_3_

**Figure S48:**^13^C NMR spectrum of**6** in CDCl_3_

**Figure S49:** DEPT-135 NMR spectrum of **6** in CDCl_3_

**Figure S50:**^1^H NMR spectrum of **12** in CDCl_3_

**Figure S51:**^13^C NMR spectrum of **12** in CDCl_3_

**Figure S52:** DEPT-135 NMR spectrum of **12** in CDCl_3_
